# Nosocomial *Plasmodium falciparum *infections confirmed by molecular typing in Medellín, Colombia

**DOI:** 10.1186/1475-2875-4-9

**Published:** 2005-02-09

**Authors:** Lina González, Jesus Ochoa, Liliana Franco, Marta Arroyave, Eliana Restrepo, Silvia Blair, Amanda Maestre

**Affiliations:** 1 Grupo Malaria, Facultad de Medicina, Universidad de Antioquia, Calle 62 #52-59, Lab 610, Medellin, Colombia; 2 Hospital A, Medellin, Colombia

## Abstract

Three cases of nosocomial malaria are reported from patients of the Internal Medicine Ward of a tertiary University teaching hospital in Medellin, Colombia. Epidemiological research, based on entomological captures, medical records review and interviews of nursery staff about patient care practices potentially involving contact with blood, were carried out. Molecular characterization of *Plasmodium falciparum *was based on the amplification of MSP1, MSP2 and GLURP genes. This method enabled confirmation of the same *P. falciparum *genotype in all three patients as well as in a fourth one (index case). The presence of nosocomial malaria was confirmed and it was concluded that the most likely source of transmission was through multi-dose preparations of heparin applied to heparin locks.

## Background

The city of Medellin is located in the north-western region of Colombia and, although malaria is endemic in the country, absence of anophelines within the city makes vector transmission of the infection impossible. However, this city, located within the Andes Mountains (6°13'N, 75°36'W) at 1,588 m above sea level (a.s.l.), is surrounded by highly endemic regions and imported cases are often referred to the University hospital for treatment. Here, three cases of nosocomial malaria, which occurred in hospitalized patients in the Internal Medicine Ward of Hospital A, are described. These patients were being treated for diverse conditions and they shared the hospital facilities during different periods. One of these patients was in the Emergency Ward at the same time as a *Plasmodium falciparum*-infected patient.

In order to confirm an association between all the cases, *P. falciparum *genomic DNA was amplified using specific primers for polymorphic streches of Merozoite Surface Antigen-1 (MSP-1), Merozoite Surface Antigen-2 (MSP-2) and Glutamate Rich Protein (GLURP), and the profile observed among the different isolates was compared.

## Case presentations

### Patient 1. Index case

A 23-year-old male woodcutter, resident in Vigia del Fuerte (6.35N, 76.53W), was referred to the hospital on 11 April 2001, diagnosed with yellow fever. Vigia del Fuerte is a highly endemic malaria region located in the Atrato River basin with a mean Annual Parasite Index (API) of 72.6 during the past five years. The patient presented 15 days with fever, headache, chills, jaundice, arthralgia, melena, dark urine and bilious vomiting. He was given a heparin lock for administration of I.V. treatment. The patient deteriorated throughout the night and died before thick smear results were available. Peripheral blood thin and thick smears confirmed the presence of *P. falciparum *asexual forms (>100,000 per μl). A post-mortem confirmed as cause of death complicated malaria. Dengue and yellow fever IgM antibodies were absent. The patient remained in the emergency ward for 18 hours prior to his death.

### Patient 2

A 19 year-old male, unemployed, resident in Angelopolis (Antioquia, 6.06°N 75.42°W), a non-malarious area, located 1,900 m a.s.l., attended the emergency service of the hospital on 10^th ^April 2001, and remained there for 24 hours before being transferred to the Internal Medicine Ward with diagnosis of systemic lupus erythematosus (SLE). In addition to symptoms and signs of SLE, he presented fever on 23 April, and received treatment with fenitoin, dipirone, ampicillin, ranitidine, ceftriazone, methilprednisolone, oxacillin, amikacin and enoxaparine (via an heparin lock). As part of the treatment for his condition, he was administered chloroquine 150 mg daily from 24^th ^April 2001. He was discharged on 19^th ^May 2001. Then, on 6^th ^June 2001, he was hospitalized with a diagnosis of a febrile syndrome. *P. falciparum *malaria was confirmed on 20^th ^June 2001 after observation of the parasite in a blood cell count. A thick smear revealed the presence of 14,440 asexual forms per μl in peripheral blood. The patient was administered quinine and sulphadoxine-pyrimethamine with good treatment response.

### Patient 3

A 46 year-old male, unemployed, resident of Santa Barbara (Antioquia, 5.57°N 75.91°W), a non-malarious area located 1,800 m a.s.l., was hospitalized in the Internal Medicine Ward on 8^th ^May with diagnosis of status asthmaticus. He was administered aminophyllin, hydrocortisone, ranitidine, enoxaparine (via a heparin lock) and was discharged on 15^th ^May 2001. He returned to the hospital on 5^th ^June 2001 complaining of fever, chills and adynamia and was admitted with diagnosis of toxic hepatitis due to the presence of dark urine, jaundice and hepatomegaly. On 6^th ^June 2001, diagnosis of complicated malaria was confirmed by observation of the parasite in a blood cell count. Later, the presence of 87,500 trophozoites of *P. falciparum *per μl was confirmed by a thick smear. He received treatment with quinine, sulphadoxine-pyrimethamine and was discharged on June 23^rd ^2001

### Patient 4

A 41 year-old male, flower-grower, resident of El Carmen del Viboral (Antioquia, 6.09°N 75.34°W), a non-malarious area located 2,150 m a.s.l.., was admitted directly to the Internal Medicine Ward of the hospital on 7^th ^May 2001 with superior vena cava syndrome (100% obstruction) and was applied a heparin lock for administration of I.V treatment. The patient was diagnosed with a mediastinal mass, compatible with lymphoma, and received treatment with dexametasone (I.V.) and radiotherapy. On June 9^th ^2001, he evidenced fever and on 9^th ^June 2001, he was diagnosed as complicated malaria by a cell blood count. A thick smear confirmed the presence of 166,440 asexual forms of *P. falciparum *per μl. He received treatment with quinine, sulphadoxine-pyrimethamine with good treatment response. However, on 1^st ^July 2001, he died as a consequence of multiple non-malaria related complications.

### Entomological captures

Search for *Anopheles *was carried out by expert personnel from the Secretariat of Health, both within the ward and in the surrounding gardens, around the time of malaria diagnosis of patients 2 and 4. These confirmed the absence of the vector in the hospital area.

### Molecular analysis

*P. falciparum *genomic DNA was extracted from whole blood collected onto filter paper or from paraffin embebbed brain tissue (for the index case). Paraffin was removed from post-morten material using xylene, followed by 2 washes with 100% ethanol. DNA was isolated by proteinase K digestion, followed by four rounds of phenol-chloroform extraction. Purified DNA was stored at -20°C until amplification.

The Region 2 of MSP-1 and the central region of MSP-2 were amplified by a nested Polymerase Chain Reaction (PCR); the region RII of GLURP was amplified by a semi-nested PCR [1]. Products obtained after the first PCR were amplified using specific primers for region 2 of MSP-1 corresponding to MAD20, K1 and RO33 allelic families, and FC27 and IC-1 for the central region of MSP-2. Briefly, PCR was carried out in a total volume of 20 μL, containing 10 mm Tris-HCl (pH 9,0 at 25°C), 50 mm KCl and 0,1% Triton X-100, 125 mM dNTPs, 0,4 units Taq DNA polymerase (Promega, Madison, WI), 1,6 mM MgCl2 and 125 nM of each primer. Initial denaturation was 5 min at 95°C, 1 min at 94°C, 2 min at 58°C annealing (all first PCR reactions and second reaction for GLURP) or at 61°C (for all the second reactions of MSP-1 and 2). This was followed by extension for 2 minutes at 72°C. This first reaction underwent 25 amplification cycles and the second 30 cycles. Positive (strains HB3, K1 and RO33) and negative controls (healthy individuals), were included.

**Figure 1:**
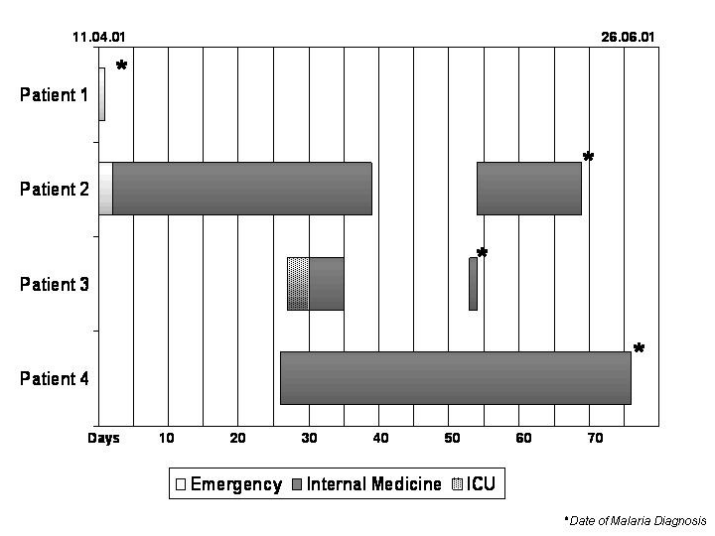
Dates, chronology of exposure, place of contact and time of malaria diagnosis of the index case and the 3 patients with nosocomial malaria. Each bar represents the location of each patient during the stay at the hospital.

Products were electrophoresed on an agarose (MetaPhor) gel (2,5% for MSP-1 and 2% for MSP-2 and GLURP), stained with ethidium bromide and visualized under ultraviolet light. Size analysis of the amplified fragments revealed identical pattern distribution for all the examined markers assayed, confirming the presence of *P. falciparum *infection by matching strains in all 4 patients (Fig 1).

## Conclusions

The presence of *P. falciparum *malaria infection in three patients without history of malaria or travel to malaria-endemic regions is described. The first of these patients (Patient 2) shared the Emergency Ward with a fatal case of falciparum malaria. The incubation period for this nosocomial infection was 12 days, however, since he was administered high doses of chloroquine, fever receded for about two weeks. Resistance of *P. falciparum *to chloroquine is highly prevalent in the Antioquia region [2], this explains the lack of efficacy of the antimalarial in eradicating parasites in this patient.

Patient 2 shared the Internal Medicine facilities with patients 3 and 4 at least for one week during May. Patient 3 evidenced an incubation period of 22 days, while in Patient 4 this was 26 days.

**Figure 2:**
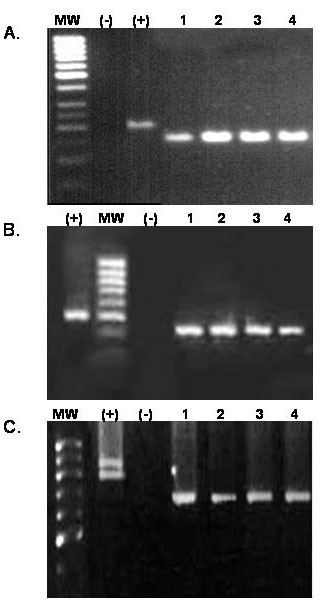
Agarose gel electrophoresis showing the size of the amplified products of the genes MSP-1 (panel A), MSP-2 (panel B) and GLURP (panel C). MW represents the molecular weight marker,(-) a negative control and (+) a positive control. The numbers are equivalent to the case number. Panel A corresponds to allelic family MAD-20 of MSP-1 showing amplification products of ~150 bp in the cases, panel B corresponds to allelic family IC50 showing amplification products of ~450 bp in the cases, panel C corresponds to GLURP showing amplification products of ~700 bp in the cases.

Since patients 3 and 4 had no contact with the index case, further analysis of the incubation periods allowed us to conclude that transmission occurred from index case to patient 2 and the latter was the source of infection for patients 3 and 4. This means that at least 2 separate episodes of contamination were involved in transmission to the different patients (see figure 2). Since entomological captures were negative for vectors and Medellin is not an area of vector transmission for malaria, parenteral contamination might be the most likely mechanism of transmission. None of the patients was administered blood before diagnosis of malaria, but all of them had at least one heparinized lock during their stay either in the Emergency or in the Internal Medicine Ward.

Transmission of malaria via heparin locks has been reported previously by other authors, the source of contamination was a multidose heparin container [3]. Although the practice of multiple dose preparations of heparin into large volume syringes to be distributed among the several patients of the ward is forbidden at this tertiary institution, it has been difficult to eradicate due to the high volume of patients and the limited economical resources of the hospital.

Other researchers have reported on the use of molecular typing of *P. falciparum *to confirm nosocomial transmission of malaria [4]. Genotyping of all 4 cases confirmed the epidemiological suspicion of nosocomial transmission. Considering the previous report on the limited genetic polymorphism of *P. falciparum *observed in the Antioquia region [5], the presence of an identical genotype of *P. falciparum *among unrelated parasites may be possible, but unlikely, since examination of these 3 different genes has been routinely used to discriminate unrelated parasites [6]. Moreover, the molecular findings together with the epidemiological characteristics contribute to confirm nosocomial transmission of the infection.

These observations are of major relevance to sanitary and health authorities, since they confirm the importance of biosafety during minor procedures, such as application of heparin to peripheral locks. In addition, it highlights the need to rule-out malaria infections in all febrile patients sharing hospital facilities with malaria patients as well as in those not responding as expected to antimicrobial therapy.

## Authors' Contributions

GL was involved in the review of clinical records, staff interviews and preparation of the manuscript. OJ coordinated the epidemiological and entomological researches. FL coordinated the microscopy diagnosis and following of the patients. AM participated on the epidemiological research. RE carried out the molecular analysis of the samples. BS, the Grupo Malaria leader, participated in the treatment and following of the patients. MA attended the patients, coordinated the clinical and molecular aspects of the research and prepared the manuscript.
